# Open‐Label Pilot Study of Interferon Gamma–1b in Patients With Non‐Infantile Osteopetrosis

**DOI:** 10.1002/jbm4.10597

**Published:** 2022-01-25

**Authors:** Andrew Nguyen, Weston P. Miller, Ashish Gupta, Troy C. Lund, Daniel Schiferl, Lok Sze Kelvin Lam, Zorayr Arzumanyan, Paul J. Orchard, Lynda E. Polgreen

**Affiliations:** ^1^ Lundquist Institute for Biomedical Innovation at Harbor‐UCLA Medical Center Torrance CA USA; ^2^ Audentes Therapeutics, An Astellas Company San Francisco, (formerly at University of Minnesota) San Francisco CA USA; ^3^ University of Minnesota Minneapolis MN USA; ^4^ Bone Diagnostics Spring Branch TX USA

**Keywords:** OSTEOPETROSIS, DISEASES AND DISORDERS OF/RELATED TO BONE, BONE QCT/μCT, ANALYSIS/QUANTITATION OF BONE, THERAPEUTICS

## Abstract

The only treatment currently available for patients with severe infantile osteopetrosis is hematopoietic cell transplantation (HCT). HCT‐related toxicity and mortality risks typically preclude its use in non‐infantile patients, and other therapies are needed for these patients who have significant disease‐related morbidity. Interferon gamma‐1b is currently approved by the U.S. Food and Drug Administration (FDA) for treatment of severe infantile osteopetrosis (autosomal recessive osteopetrosis [ARO]). However, little is known about the effects of interferon gamma‐1b in non‐infantile osteopetrosis. Thus, this pilot study aimed at testing the safety and tolerability of interferon gamma‐1b in patients with non‐infantile osteopetrosis and assessing the clinical effects. We performed a 12‐month, open‐label, multi‐center pilot study involving patients >1 year‐old diagnosed radiographically with osteopetrosis. Patients were initiated on interferon gamma‐1b subcutaneously 15 μg/m^2^ three times weekly, to be titrated over 3 weeks to a goal of 100 μg/m^2^ three times weekly. The primary aim was safety and tolerability. The secondary aims were to assess changes in peripheral quantitative computed tomography (pQCT), dual‐energy x‐ray absorptiometry (DXA) bone mineral density (BMD) Z‐scores, bone biomarkers, and quality‐of‐life (QOL) measures. Four of the five participants enrolled withdrew from the study between 3 and 9 months due to intolerability of interferon gamma‐1b–related flu‐like symptoms. The last participant completed the study with the addition of prednisone on days of interferon gamma‐1b administration. DXA and pQCT outcomes were stable over 6–12 months, and there were no clear trends in bone biomarkers or QOL measures. No serious drug‐related adverse events were reported during this study. Interferon gamma‐1b was only tolerable in one of five participants with the addition of prednisone. The stabilization of BMD and other measures of bone health during this study suggest possible positive effects of interferon gamma‐1b on osteopetrosis; however, additional data are needed before conclusions on treatment efficacy can be made. © 2022 The Authors. *JBMR Plus* published by Wiley Periodicals LLC on behalf of American Society for Bone and Mineral Research.

## Introduction

Osteopetrosis is a rare inherited metabolic bone disease characterized by impaired osteoclast function resulting in defective bone resorption and generalized high bone mass and bone mineral density (BMD). In patients with severe disease, this high bone mass compromises bone marrow space leading to marrow failure and frequent infections, along with hepatosplenomegaly from extramedullary hematopoiesis. Visual defects may result due to impingement of cranial nerves. Other severe sequelae can include osteomyelitis, hypocalcemia and hypocalcemic seizures, “brittle bones” causing an increased fracture risk, or osteopetrotic rickets in childhood.^(^ ^1^ ^)^

Prior to the availability of categorizing disease based on genotype, three clinical phenotypes were described as (i) severe infantile, (ii) intermediate, and (iii) adult onset. More recently, phenotypes can be defined based on two distinct genotypes: (i) autosomal recessive osteopetrosis (ARO), which can be further broken down to subtypes of severe ARO—diagnosed in infancy—and attenuated ARO—which may present in childhood and adolescence; and (ii) autosomal dominant osteopetrosis (ADO2), with a wide spectrum of disease and more protracted clinical course.^(^ ^2^ ^)^ Medical literature still references the intermediate osteopetrosis phenotype, but advances in genetics demonstrate that this is a mixed group with patients with attenuated ARO or more severe forms of ADO2. Disease severity is highly variable.

There are currently no curative therapies for individuals with non‐infantile osteopetrosis (ADO2 or attenuated ARO). The consensus guidelines developed by the Osteopetrosis Working Group recommend symptom based supportive therapy for disease complications.^(^ ^3^ ^)^ The only treatment for individuals with severe forms of osteopetrosis is hematopoietic cell transplantation (HCT); however, survival in patients is only around 55%–85% for attenuated ARO and even lower for ADO2.^(^ ^4^, ^5^ ^)^ Therefore, this treatment is only indicated in select individuals with life‐threatening complications of their disease. Clearly, additional treatments for osteopetrosis are needed for individuals who are not candidates for HCT or where the risk associated with HCT outweighs the potential benefit.

Interferon gamma is a naturally occurring cytokine that has been shown to have anti‐microbial and anti‐viral immunomodulatory effects,^(^ ^6^ ^)^ and is a potent stimulator of superoxide anion production, which in turn promotes the formation and activation of osteoclasts.^(^ ^7^ ^)^ Interferon gamma‐1b has been shown to increase osteoclast size, tartrate acid phosphatase staining, and superoxide anion production in cell culture from patients with osteopetrosis.^(^ ^8^ ^)^ In addition, osteopetrotic mice have increased marrow space following treatment with interferon gamma‐1b.^(^ ^9^ ^)^

There have been two previous studies of interferon gamma‐1b in a small group of individuals with ARO.^(^ ^10^, ^11^ ^)^ In one study, Key and colleagues^(^ ^11^ ^)^ followed 14 patients with ARO receiving therapeutic interferon gamma‐1b for osteopetrosis. After 6 months of therapy, all 14 ARO patients showed significant decreases in trabecular bone area as assessed as a percentage of total bone biopsy area, from 55.3% ± 15.0%, to 33.2% ± 18.0% (*p* = 0.002). This improvement was additionally found to be sustained in the 11 patients that remained in the study to 18 months, at 34.6% + 3.5% (*p* = 0.02). Lumbar spine (L_1_–L_4_) BMD measured by dual‐energy X‐ray absorptiometry (DXA) decreased by 14% at 6 months and 17% at 18 months. Hemoglobin concentration increased on average from 7.5 to 10.5 g/dL at 18 months (*p* = 0.05), while superoxide generation by granulocyte macrophage colonies as measured by nitroblue tetrazolium reduction improved from 0.26 to 0.41 optical density units (*p* < 0.001).^(^ ^10^ ^)^ These studies resulted in U.S. Food and Drug Administration (FDA) approval of interferon gamma‐1b for ARO but not for ADO2 because of the young age of the participants. These studies were performed prior to the identification of the genetic basis of recessive osteopetrosis, including the TCIRG1 and CLCN7 genes, of which CLCN7 is also implicated in dominant disease. Based on these studies, and the relatively high fatality rate of hematopoietic stem cell transplant (HSCT) in patients with non‐infantile disease, we conducted a 12‐month open‐label pilot study of interferon gamma‐1b in patients with non‐infantile (severe ARO or ADO2) osteopetrosis.

## Patients and Methods

### Trial design

This study was a 12‐month open label pilot study of the safety and tolerability of interferon gamma‐1b in individuals with non‐infantile osteopetrosis. Patients diagnosed with osteopetrosis based on radiographic findings, who were ≥1 year of age, and could travel to one of two study centers were included in the study if they had one or more of the following: (i) hemoglobin <12 g/dL, not related to iron deficiency; (ii) absolute neutrophil count <1000 neutrophils/μL unsupported with cytokines; (iii) platelet count <50,000 cells/μL; (iv) history of impaired bone healing; and (v) one or more serious infections over prior year, defined as requiring hospitalization and/or intravenous (iv) antibiotics. Patients were excluded from participation in the study for any of the following: (i) history of HCT; (ii) pregnant or breastfeeding; (iii) known or suspected allergy to interferon gamma‐1b or related products; (iv) participation in any simultaneous therapeutic study that involved an investigational drug or agent within 4 weeks of study enrollment; (v) alanine aminotransferase (ALT) >3× upper limit of normal; and (vi) thalassemia or other hemoglobinopathy due to potential for inhibition of erythropoiesis and/or increased red blood cell destruction (added after worsening anemia in one participant with thalassemia).^(^ ^12^, ^13^, ^14^, ^15^ ^)^

The primary endpoint of this pilot study was to determine the safety and tolerability of interferon gamma‐1b in patients with non‐infantile osteopetrosis. Specifically, it involved the ability continue on treatment at the goal dose for 12 months following enrollment. The secondary endpoints of this study were to obtain preliminary efficacy data on changes in bone density and other measures of bone health, markers of bone formation and resorption, physical function, and quality‐of‐life.

### Intervention

To minimize side effects, patients were to receive an escalating dose of interferon gamma‐1b (ACTIMMUNE; Horizon Therapeutics, Dublin, Ireland) over the first 4 weeks of the study: initial dose of interferon gamma‐1b subcutaneous 15 μg/m^2^ three times weekly for week 1, then escalated to 30 μg/m^2^ three times weekly for week 2, 50 μg/m^2^ three times weekly for week 3, then up to the target dose of 100 μg/m^2^ in week 4. Doses were decreased and titrated to tolerability on a case‐by‐case basis at the discretion of the site principal investigator (PI) to manage subsequent drug‐related adverse events. Once it was determined that interferon gamma‐1b was intolerable, a protocol amendment was made to add prednisone 20 mg by mouth on the days of injections (2 days per week) to prevent the flu‐like reaction.

### Human subjects approval

This study was conducted in accordance with the Declaration of Helsinki and was approved by the Institutional Review Board (IRB) at each institution. Prior to enrollment, all subjects or parents for subjects under the age of 17 years provided written consent after appropriate discussion of the risks and benefits of participation. Assent was obtained from participants 7–17 years old. The trial is listed on ClinicalTrials.gov identifier NCT02666768. A Data and Safety Monitoring Plan approved by the local IRBs was followed that included an individual Data Safety Monitor with relevant expertise who reviewed prespecified data every 6 months.

### Assessments

To minimize ascertainment bias, a Manual of Operations and standardized case report forms were used to collect samples and outcome measures consistently at each site. However, because the one participant enrolled at site 2 withdrew before month 3 assessments, all data we present herein were collected at one site.

Participants were contacted monthly to obtain data on adverse events and recorded in the REDCap (https://www.project-redcap.org/) database.

Radiographic assessments were performed using peripheral quantitative computed tomography (pQCT) (XCT 2000; Stratec Medizintechnik, Pforzheim, Germany) to assess change in volumetric bone mineral density (vBMD) at the tibia 3% and 38% sites. The radius 3% and 38% sites were added after participant 3 enrolled due to inability to obtain tibia measurements in this participant. With osteopetrosis, the trabecular and marrow areas fill in with bone so a traditional pQCT analysis does not work. A complete description of the pQCT analysis approach used is included as Supplemental Methods.

Bone effects were also measured using DXA (Hologic Discovery A; Hologic, Marlborough, MA, USA) to assess areal bone mineral density (aBMD) *Z*‐scores.

Patient reported outcomes were collected using two different surveys: Child Health Questionnaire ‐ Parent Form 50 (CHQ‐PF50; healthactchq, Inc., Boston, MA, USA) and the RAND Medical Outcomes Study Short Form‐36 (SF‐36; RAND Corporation, Santa Monica, CA, USA) for participants <18 years and ≥18 years, respectively.

Blood and urine samples were collected in the morning after a minimum 8‐hour fast, immediately processed, frozen, and stored at −80°C. All biomarker samples were run in one batch at the end of the study in the TransGenomics Laboratory at the Lundquist Institute (Torrance, CA, USA). C‐telopeptide (CTX; MyBioSource, Inc., San Diego, CA, USA; MBS2607151) and total Procollagen Type I Intact N‐terminal Propeptide (P1NP; MyBioSource, Inc.; MBS165070) were measured by enzyme‐linked immunosorbent assay (ELISA) using kits from MyBiosource, Inc.. Receptor activator of nuclear factor κΒ ligand (RANKL; ab213841) and osteoprotegerin (OPG; ab189580) were measured by ELISA using kits from Abcam (Cambridge, MA, USA).

Safety laboratories were measured at Quest Diagnostics (Secaucus, NJ, USA).

### Statistical analysis

Descriptive statistics are presented as mean ± standard deviation (SD) for continuous variables, and as frequency and percent for nominal variables. Change in outcomes over time was determined using GEE analysis (STATA 14.2; StataCorp, LLC, College Station, TX, USA). For all analyses, *p* values are presented; however, given the small sample size, significance, or lack thereof, should be interpreted with caution.

## Results

### Study population

We enrolled five participants between April 2016 and March 2018, with a clinical diagnosis of osteopetrosis based on characteristic radiographic findings: four with ADO2 and one with attenuated ARO. Lumbar spine aBMD *Z*‐score by DXA at baseline ranged from +4.7 to +18.9, and total body from +4.2 to +13.5. It was not a requirement to have genetic confirmation of osteopetrosis, though of the five participants, four had genetic confirmation.

Baseline characteristics are shown in Table 1. No participants were taking calcitriol or glucocorticoids for the 12 months prior to enrollment or during the trial, except for the last participant for whom prednisone was prescribed by the study physician at month 3. Baseline laboratory values for parathyroid hormone, total calcium, absolute neutrophil count, platelet count, and liver and kidney function were all normal. All 25‐hydroxy vitamin D levels were >20 ng/mL for the duration of the study. Baseline hemoglobin was low for all participants, ranging from 8.2 to 10.6 g/dL in females, and 13.2 g/dL in one male participant. Urine calcium/creatinine ratio was <0.18 mg/mg in all participants. No participant developed anti‐drug antibodies.

**Table 1:** Description of Participants

Participant	Age (years)	Sex	Genotype	Site	Diagnosis	Race	Eligibility for study entry
1	22	F	CLCN7 c.857G>A; Arg286G	1	ADO2	Black	Anemic, history of impaired bone healing, 1 or more serious infection over prior year
2	59	F	CLCN7 c.2250+1G>T, heterozygous, intron 23	1	ADO2	White	Anemic, history of impaired bone healing
3	48	M	Unknown^a^	1	ADO2	Other or mixed	History of impaired bone healing
4	36	F	CLCN7 c637C>T; Leu213Phe	2	ADO2	White	Anemic, history of impaired bone healing, sustained a fracture over the last year
5	15	F	2 VUS: TCIRG c.1249G>A and c.1735G>A	1	ARO	White	Anemic, history of impaired bone healing, sustained a fracture over the last year

ADO2 = autosomal dominant osteopetrosis type 2; ARO = autosomal recessive osteopetrosis; CLCN7 = chloride voltage‐gated channel 7; F = female; M = male; TCIRG = T cell immune regulator 1; VUS = variant of unknown significance.

^a^ Clinical diagnosis of ADO2 was made by the participant's physician based on age and radiographs prior to enrollment in the study.

All data, besides safety and tolerability data, are from four participants at a single site because one participant at a second site withdrew prior to their month 3 visit.

### Tolerability

Four of the five participants withdrew from the study between 3 and 9 months due to the intolerability of interferon gamma‐1b–related flu‐like symptoms. No participant tolerated the goal dose of 100 μg/m^2^ for the intended 12‐month duration of the study. The last subject enrolled was treated with prednisone 20 mg (0.3 mg/kg/dose) orally on days of interferon gamma‐1b administration starting at month 3, and that resulted in tolerance of a maximum dose of 60 μg/m^2^ subcutaneously three times weekly for the duration of the study (12 months).

Three of the five subjects did not tolerate the rate of initial dose escalation over the first 4 weeks of the study, and one never tolerated more than 30 μg/m^2^. Dose adjustments were made at the discretion of the site PI with the goal to achieve the highest tolerable dose. Specifics for each participant follow:

Participant 1: Dose decreased from 100 μg/m^2^ to 50 μg/m^2^ (1.0 μg/kg) between months 2 and 3 due to flu‐like symptoms and discontinued at month 5 due to persistent flu‐like symptoms.

Participant 2: Tolerated a dose of 100 μg/m^2^ (2 μg/kg) until month 8 then withdrew due to flu‐like symptoms.

Participant 3: Fluctuating tolerability throughout study participation. Dose was adjusted frequently for the 9‐month duration the patient continued in the study due to joint pain and flu‐like symptoms. Dosing ranged between 7.6 μg/m^2^ and 30 μg/m^2^ (0.2 μg/kg), with the majority of doses falling within the 15–25 μg/m^2^ range. Subject withdrew at month 9.

Participant 4: Tolerated 4‐week dose escalation schedule up to 100 μg/m^2^, then dose decreased to 50 μg/m^2^ at week 5 and to 30 μg/m^2^ at week 7 both due to flu‐like symptoms. Continued this dose for 2 weeks, then attempted to titrate dose up again over 4 weeks. Reached dose of 43.75 μg/m^2^ (0.7 μg/kg), at which time the subject withdrew from the study.

Participant 5: Dose was titrated per protocol up to 60 μg/m^2^ (0.7 μg/kg), which was the maximum tolerated dose with the addition of prednisone at month 3.

### Bone

**Fig. 1:**
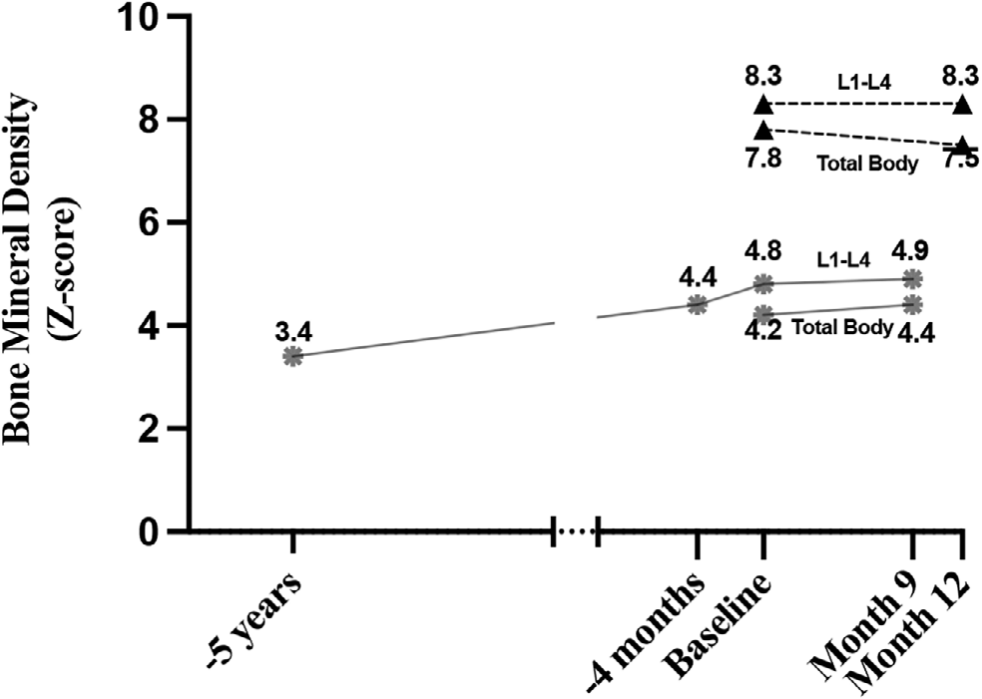
Change in DXA *Z*‐scores over time in two participants: dashed lines with triangle is participant 5 (ARO) and solid lines with star is participant 3 (ADO2). Data are only available for two participants due to withdrawal of all other participants before month 9. DXA data prior to baseline were available from one participant's medical records and so included to show change over time, but data prior to study enrollment were collected on different machines than the one used in the trial.

The change in DXA *Z*‐scores over time from two of the participants is shown in Fig. 1. The *Z*‐scores over the duration of the trial were generally stable. Lumbar spine DXA *Z*‐scores (from a different DXA than the one used for the study) from prior to study enrollment were available in one participant; these show a gradual increase in *Z*‐score over the 5 years prior to baseline measurement. Lumbar spine (L_1_–L_4_) BMD increased by 1% in participant 3 and 3% in participant 5. Total body BMD increased by 2% and 1%, respectively.

**Fig. 2:**
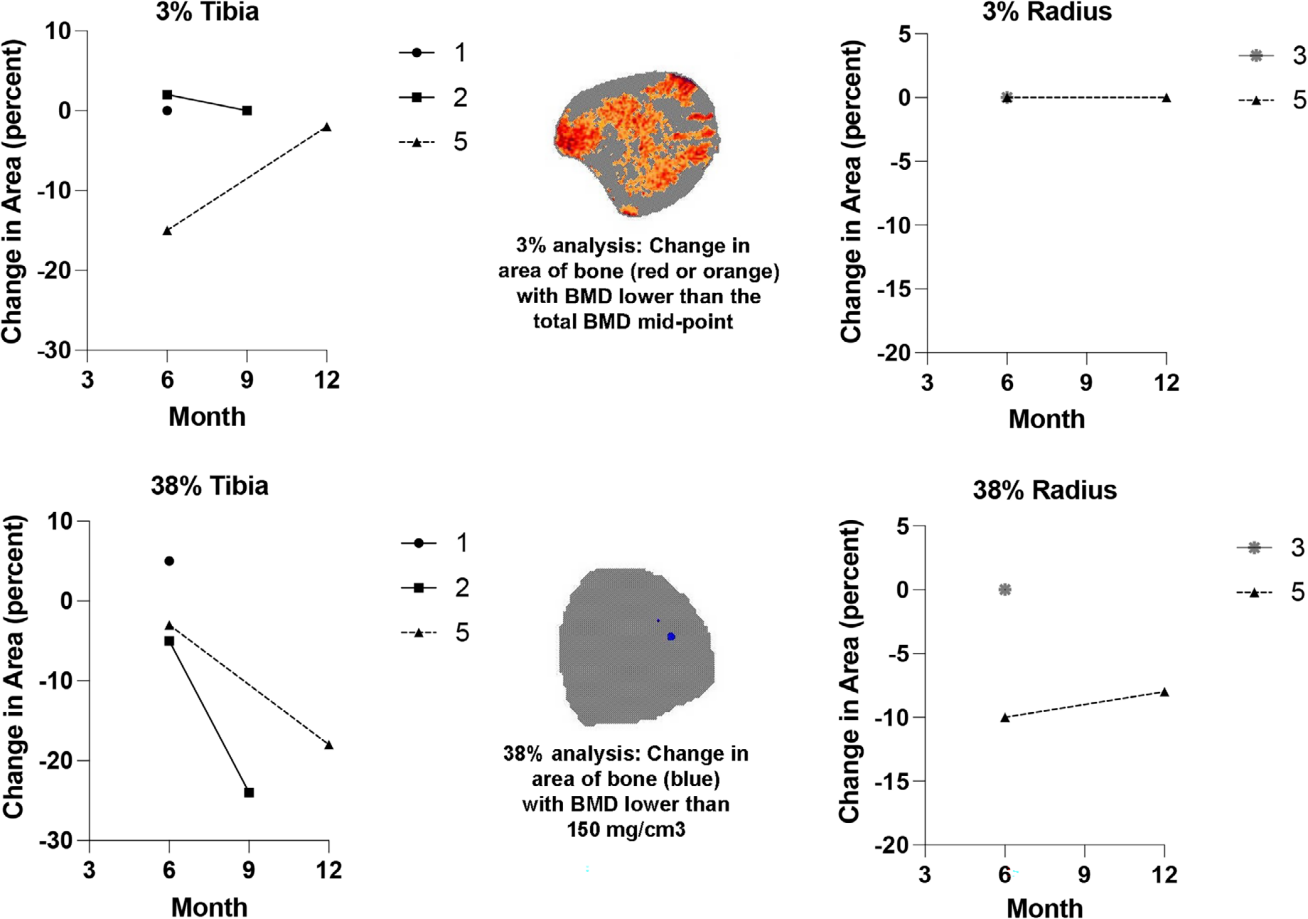
Percent change in the area of bone in the tibia and/or radius with BMD below the total BMD mid‐point at the 3% site and below 150 mg/cm^3^ at the 38% site measured by pQCT. Missing data are because the radius was added to the study after we were unable to obtain tibia measurements in participant 3 due to pQCT gantry size. Details of the analysis are available in Supplement Document 1.

With osteopetrosis the trabecular and marrow areas fill in with bone so a traditional pQCT analysis does not work well. Our goal was to find if the bone was changing, and if so where, so we used an approach that quantified the area of bone with a BMD below the total BMD mid‐point for the 3% site and the area of bone with a BMD <150 mg/cm^3^ at the 38% site. These changes in area are shown in Fig. 2.

**Fig. 3:**
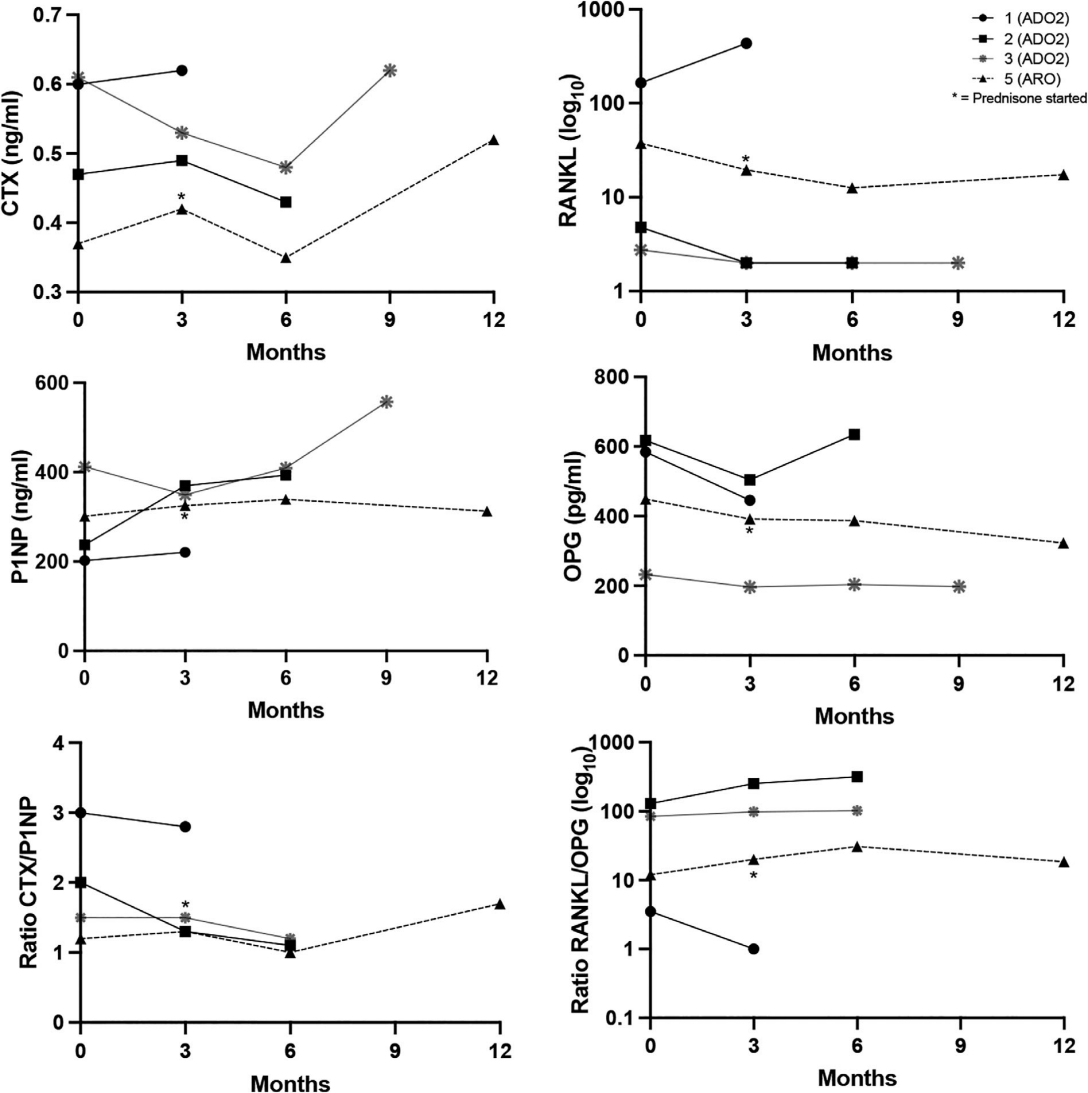
Changes in markers of bone formation (P1NP, OPG) and bone resorption (CTX, RANKL), along with ratios of bone resorption to bone formation over 3–12 months of interferon gamma‐1b treatment. Dashed line is participant 5 (ARO) and solid lines are participants 1–3. Participant 4 was not included because only baseline samples were collected. *Indicates where prednisone 20 mg on day of injections was started in the one participant with severe ARO.

Changes in biomarkers over the course of the trial are shown in Fig. 3. CTX and RANKL were generally stable or decreasing from baseline to month 6, and then CTX increased in the remaining two participants after month 6. P1NP increased in two participants and was stable in two. OPG had a slight trend downward in three of four participants. The ratio of bone resorption to bone formation was generally stable except for a downward trend in two participants.

### RAND SF‐36

The RAND SF‐36 survey outcomes measuring quality of life are reported in Table 2 for the four participants with ADO2; the participant with ARO was too young for this survey and so completed the CHQ‐PF50 with a parent instead. Although there were no differences demonstrated in physical functioning, role limitations due to physical limitation, emotional problems, energy, emotional well‐being, social functioning, pain, or general health, it was noted that RAND SF‐36 Survey outcomes demonstrated worsening scores in physical function and pain, possibly as a side effect of the interferon treatment. However, without a control group of patients only receiving current standard of care, the true effect on these outcomes cannot be assessed. It is possible that interferon gamma‐1b prevented steeper decline in these scores compared to current therapies available.

**Table 2:** Change in Medical Outcomes Study SF‐36

	Estimated change over time (95% CI)
Scale	*n* = 4 (all ADO2)
Physical functioning	−0.5 (−1.2, 0.1)
Role limitations due to physical function	−0.2 (−0.7, 0.3)
Role limitations due to emotional problems	0.1 (−0.2, 0.5)
Energy/fatigue	0.1 (−0.4, 0.6)
Emotional well‐being	0.1 (−0.3, 0.6)
Social functioning	−0.2 (−0.9, 0.5)
Pain	−0.8 (−2.0, 0.3)
General health	0.28 (−0.04, 0.59)

ADO2 = autosomal dominant osteopetrosis type 2; CI = confidence interval; SF‐36 = Short Form‐36.

### Adverse effects

All subjects had one or more adverse events. The most commonly reported adverse event was flu‐like symptoms, and this effect was the main limiting factor in this study leading to withdrawal of four of five participants. No serious drug‐related adverse reactions were reported during this study. A summary of adverse events is shown in Supplemental Table S1.

## Discussion

In this pilot study to explore the use of interferon gamma‐1b in severe ADO2 and attenuated ARO, continuation of the drug was found to be intolerable due to flu‐like side effects. However, we learned that the addition of prednisone prevented the intolerable side effects in one patient in whom prednisone was administered concurrently. The patient who completed the entire duration of the study with glucocorticoids as an adjunctive therapy provides some evidence supporting the tolerability of interferon gamma‐1b as a potential therapeutic agent for this rare disease that is otherwise lacking in treatment options. We believe the data obtained through this pilot study supports pursuing further evaluation of interferon gamma‐1b in combination with glucocorticoids in a larger trial with the goal of elucidating not only additional safety and tolerability data, but also clinical efficacy.

Interferon gamma is produced primarily by T‐lymphocytes, natural killer cells, and natural killer T‐lymphocytes. The cytokine has many effects on both host defense and immune regulation—including antiviral, antimicrobial, and antitumor activity. Conversely, aberrant interferon gamma expression has been associated with a number of autoinflammatory and autoimmune diseases. As a result of this broad range of effect, interferon gamma has been utilized clinically as primary, adjunctive, and experimental treatment for a similarly broad array of other medical conditions, including cancer, tuberculosis, hepatitis, chronic granulomatous disease, and atopic dermatitis.

The major limiting factor in our study was the severe flu‐like reaction experienced by all participants. Glucocorticoids have been used as an adjunct with interferon treatments to improve tolerability in several different health conditions. For example, low‐dose prednisone plus interferon beta‐1b improved flu‐like symptoms compared to interferon beta‐1b alone in a study of 71 patients with multiple sclerosis.^(^ ^16^ ^)^ In another study of interferon beta‐1b treatment of patients with multiple sclerosis, low‐dose steroids decreased the percentage of macrophage and B‐lymphocytes producing IL‐6.^(^ ^17^ ^)^ This fits with other studies that have demonstrated IL‐6 to be a proinflammatory cytokine with a central role in mediating fever and acute phase response. In the murine model, injection of lipopolysaccharide into a sterile, subcutaneous air pouch evoked a body temperature increase that was accompanied by a significant increase in concentrations of IL‐1 and IL‐6 within the pouch, but only IL‐6 in circulation and cerebrospinal fluid, providing some evidence supporting IL‐6 as the principal endogenous circulating pyrogen responsible for activating central nervous system (CNS) mechanisms of fever and inflammation.^(^ ^18^ ^)^

Although flu‐like side effects were attributed as the primary cause of withdrawal for four of five participants in this study, other adverse effects also played a role. Among the side effects contributing to withdrawal was pain exacerbation. There is supporting research elucidating the mechanism of pain, specifically neuropathic pain, as mediated by interferon gamma. Naive spinal microglia express a receptor for interferon gamma based on a murine model and stimulating this receptor converts the microglia into activated cells that produced a long‐lasting pain sensitivity as measured using a behavior assay for tactile allodynia.^(^ ^19^ ^)^ Rats exposed to interferon gamma demonstrated a decreased paw withdrawal threshold to mechanical stimulation, with increased sensitivity peaking between 2 and 3 days, lasting at least 10 days after administration, and demonstrating a dose‐dependent relationship. Conversely, in a parallel experiment in which interferon gamma receptors were ablated, the animal model demonstrated an impaired pain response at all time points, without affecting the baseline mechanical sensitivity or paw withdrawal response. These findings in combination suggest the importance of interferon gamma and its associated receptor in the role of mediating signaling for converting spinal microglia to the activated phenotype to subsequently produce the pain response.

Another potential adverse effect noted within this study was the action of interferon gamma in the setting of thalassemia, as demonstrated by a participant with β‐thalassemia who developed worsening anemia that resolved after discontinuation of interferon gamma‐1b. Prior studies support a causal relationship between interferon gamma and worsening anemia. An early suggested mechanism based on cell culture studies postulated that exposure to interferon gamma causes hematopoietic suppression through inhibition of cell cycle progression and induction of apoptosis of CD34+ cells^(^ ^13^ ^)^ resulting from increased Fas antigen expression on CD34+ cells.^(^ ^12^ ^)^ More recently, it was found that interferon gamma provokes hematopoietic stem cells to favor myeloid over erythrocyte differentiation, and over self‐renewal, thus leading to depletion of the hematopoietic pool and subsequently worsening anemia.^(^ ^20^, ^21^ ^)^ In patients with thalassemia, interferon gamma, among other cytokines, is associated with impaired reticulocyte maturation.^(^ ^15^ ^)^

Because our study had no control group and natural history data are unavailable, we need to use prior studies of interferon gamma‐1b in patients with osteopetrosis^(^ ^10^, ^11^, ^22^ ^)^ to assist with data interpretation. In the Key and colleagues^(^ ^10^, ^11^ ^)^ studies, they reported that lumbar spine BMD by DXA decreased by 14% at 6 months and 17% at 18 months. Lumbar spine BMD in our two participants with DXA repeated at 9 months (ADO2) and 12 months (ARO) was 1% and 3%, respectively; change in total body BMD by DXA was 2% and 1%, respectively. In addition, there was no improvement in hemoglobin in any of our participants (data not shown)^(^ ^11^ ^)^, unlike the Key and colleagues study where hemoglobin concentration increased from 7.5 to 10.5 g/dL at 18 months. One explanation for this is the older age of our participants (age 15–59 years) compared to those in the Key and colleagues^(^ ^10^, ^11^ ^)^ studies (1 month to 11 years) because the older individuals would be expected have less marrow space and less metabolically active bone. Interferon gamma‐1b was less tolerable in our population compared to the younger group in the prior studies. We speculate that this may be at least in part due to the presence of chronic pain in all our participants, which the flu‐like side effects of interferon gamma‐1b seemed to worsen. The younger children tolerated doses of 1.5 μg/kg, which is approximately twice as high as the maximum tolerated dose in all but one of our participants.

More recently, Imel and colleagues^(^ ^22^ ^)^ conducted a 14‐week, open label, pilot study, utilizing both tolerability and a fasting C‐telopeptide <25% above baseline to guide dose titration to a goal of 100 μg/m^2^ three times a week. The study recruited nine adult patients and three pediatric patients with ADO2. Similar to our study, they found no clear changes in biochemical markers of bone resorption or formation, or most quality of life scales as measured with the SF‐36 instrument. The exception to this was a potential worsening in mental health, postulated to be due to symptoms caused by treatment administration or patient perception of overall disease burden. Bone density outcomes were not evaluated due to the short duration of the study.

Limitations of this study include the small sample size, heterogeneity of the population, and difficulty obtaining endpoint measurements due to high withdrawal rate, all resulting in limited data for interpretation. These limitations are common in rare disease clinical research, and our protocol augmented this by focusing on a specific subset of individuals with osteopetrosis who had signs of bone marrow failure. However, when we started this study there were no data to help guide clinicians on whether interferon gamma‐1b should be tried in patients with non‐infantile osteopetrosis, and there continues to be very limited data. Unfortunately, the multiple limitations of this study negate the ability to make conclusions about the efficacy of interferon gamma‐1b as treatment for non‐infantile osteopetrosis (ADO2 or attenuated ARO). But despite these limitations, this study provides information on the longest follow‐up of treatment with interferon gamma‐1b in this population and identified an approach to make the treatment tolerable.

## Conclusion

In conclusion, we have clearly shown that interferon gamma‐1b alone is intolerable to individuals with non‐infantile osteopetrosis due to flu‐like side effects. We discovered that the addition of prednisone on the days of interferon gamma‐1b administration mitigated the intolerable side effects. Interpretation of the outcomes included in this study would be greatly facilitated if more comprehensive natural history data regarding the expected change on the assessed measures were available. For now, we cannot conclude whether the overall lack of change in DXA *Z*‐scores and pQCT BMD measurements is a positive effect or no effect, although we would expect the BMD would increase over time without intervention. To obtain a definitive answer to the question of whether interferon gamma‐1b provides clinically significant benefits to individuals with non‐infantile osteopetrosis, another trial will be needed testing the effects of interferon gamma‐1b with adjunctive prednisone treatment.

## Conflict of Interests

The content of this manuscript is solely the responsibility of the authors. WPM is a full‐time employee of Audentes Therapeutics, and Astellas Company; he was employed by the University of Minnesota at the time of data collection. There are no other relevant conflicts of interest to report.

### Peer Review

The peer review history for this article is available at https://publons.com/publon/10.1002/jbm4.10597.

## Supporting information


**Appendix S1**: Supporting Information

**Appendix S2**: Supporting Information

**Table S1.** Summary of reported adverse events
