# Supplementary material for: Open‐Label Pilot Study of Interferon Gamma–1b in Patients With Non‐Infantile Osteopetrosis
Source: JBMR Plus. 2022 Jan 25;6(3):e10597. doi: 10.1002/jbm4.10597 (PMC8914146; doi:10.1002/jbm4.10597)

**Supplemental Methods - pQCT Analysis Methods (Developed by Dan Schiferl, Bone Diagnostic LLC)**

With osteopetrosis the trabecular and marrow areas fill in with bone so a traditional pQCT analysis does not work well. The goal is to find where and if the bone is changing so a different approach to analysis was needed.

One problem is visually the images can look like a solid cortical bone if you’re using a standard color palette. In order to visualize the bone density a new palette needs to be created. The image on the left is using the standard 960123 palette that displays any density above 800 mg/cm^3^ as white. On the right the palette was stretched so densities up to 1500 g/cm^3^ have color. This new palette allows the user to see differences and possibly guide us to any changes in the bone.


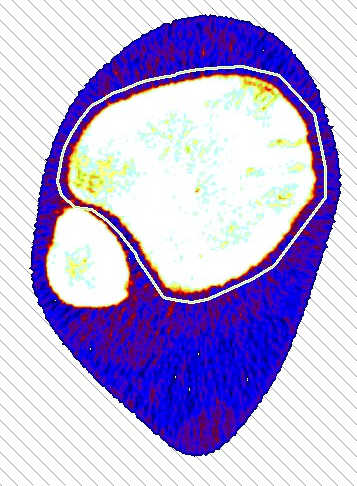

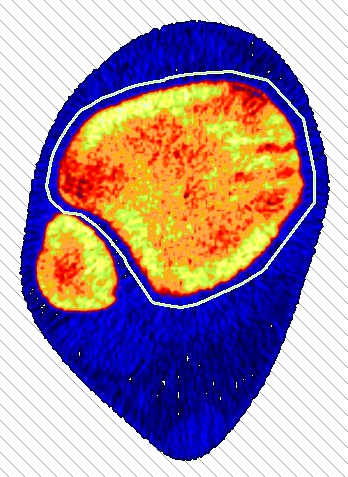


**Tibia and Radius 3% Analysis**

- **50% split of most dense vs. least dense**
  - Contour mode 3 with a threshold of 169 mg/cm^3^ which defines the periosteal edge.
  - Peel mode 20 with a 50% of total bone density: This peel mode is a way to separate trabecular bone based on a percentage setting. It works by taking all the voxel densities and calculating the mid-point (50%) density of the bone. It then reports the area of bone with a BMD below this mid-point density. With this mode the area is fixed to 50% so any change in density will be seen.


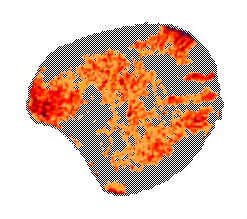


The orange and red area defines bone that is below the mid-point bone density

The gray area defines bone that is above the mid-point bone density

**Tibia and Radius 38% Analysis**

- **Special endosteal analysis using Calcbd**
  - Special ROI is drawn inside the cortical bone
  - Contour mode 1 with a threshold of -100
  - Peel mode 2 with a threshold of 150 mg/cm^3^

The blue area defines any bone and marrow with BMD lower than 150 mg/cm^3^


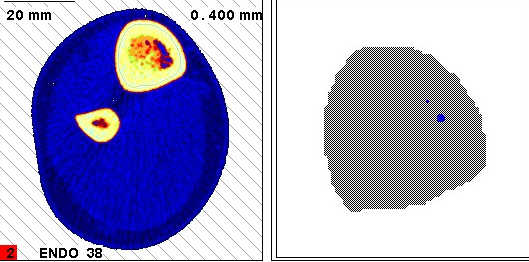

Supplement: Supplementary file 1 — Appendix S1: Supporting Information [file JBM4-6-e10597-s002.docx]
