# Supplementary material for: Open‐Label Pilot Study of Interferon Gamma–1b in Patients With Non‐Infantile Osteopetrosis
Source: JBMR Plus. 2022 Jan 25;6(3):e10597. doi: 10.1002/jbm4.10597 (PMC8914146; doi:10.1002/jbm4.10597)
Supplement: Supplementary file 2 — Appendix S2: Supporting Information [file JBM4-6-e10597-s003.docx]

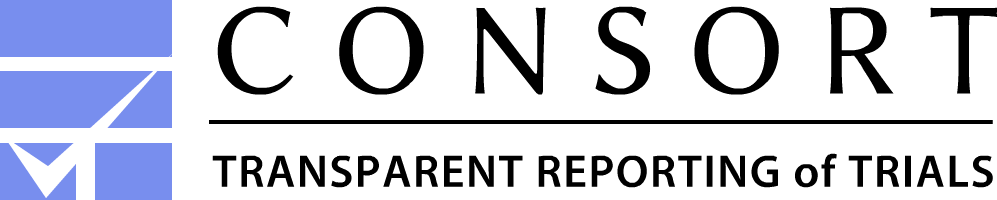


**CONSORT 2010 Flow Diagram**

## Enrollment

Lost to follow-up (give reasons) (n=0)

Discontinued intervention (give reasons) (n=0)

1. Intolerable flu-like symptoms
2. Intolerable flu-like symptoms
3. Intolerable flu-like symptoms
4. Intolerable flu-like symptoms

## Follow-Up

Allocated to intervention (n=5)

♦ Received allocated intervention (n=5)

♦ Did not receive allocated intervention (give reasons) (n=0)

## Analysis

Analysed (n=5)
♦ Excluded from analysis (give reasons) (n=0)

Excluded (n=0)

Assessed for eligibility (n=5)
