# Supplementary material for: Open‐Label Pilot Study of Interferon Gamma–1b in Patients With Non‐Infantile Osteopetrosis
Source: JBMR Plus. 2022 Jan 25;6(3):e10597. doi: 10.1002/jbm4.10597 (PMC8914146; doi:10.1002/jbm4.10597)
Supplement: Supplementary file 3 — Table S1. Summary of reported adverse events [file JBM4-6-e10597-s001.docx]

| **Supplemental Table 1. Summary of Reported Adverse Events** | | |
| --- | --- | --- |
| System Organ Class  Adverse Event | Incidence  n (%) | Events  n (rate) |
| Hematologic disorders  neutropenia  anemia  thrombocytosis | 2 (40)  2 (40)  1 (20) | 3 (0.6)  2 (0.4)  1 (20) |
| Cardiac disorders  hypertension | 1 (20) | 1 (0.2) |
| Ear and labyrinth disorders  foreign body | 1 (20) | 1 (0.2) |
| Endocrine disorders  hyperglycemia  hypokalemia  hyperparathyroidism | 2 (40)  1 (20)  1 (20) | 2 (0.4)  1 (0.2)  1 (0.2) |
| Gastrointestinal disorders  abdominal pain  dyspepsia  nausea | 2 (40)  1 (20)  3 (60) | 3 (0.6)  1 (0.2)  3 (0.6) |
| General disorders  fatigue  insomnia  flu-like symptoms  Injection site reactions (bruise,  redness) | 4 (80)  1 (20)  5 (100)  3 (60) | 5 (1.0)  1 (0.2)  13 (2.6)  11 (2.2) |
| Infections  gingival/dental abscess  osteomyelitis  sinusitis/sinus pain/sinus congestion  pharyngitis  cellulitis  urinary tract infection | 1 (20)  1 (20)  2 (40)  1 (20)  1 (20)  1 (20) | 2 (0.4)  1 (0.2)  4 (0.8)  1 (0.2)  1 (0.2) 2 (0.4) |
| Musculoskeletal  bone pain - generalized  fracture  joint pain  myalgia  muscle cramp | 2 (40)  2 (40)  2 (40)  3 (60)  1 (20) | 2 (0.4)  3 (0.6)  4 (0.8)  5 (1.0)  1 (0.2) |
| Nervous system disorders  headache  neuralgia  poor concentration | 4 (80)  1 (20)  1 (20) | 8 (1.6)  1 (0.2)  1 (0.2) |
| Psychiatric disorders  mood lability  insomnia | 1 (20)  1 (20) | 1 (0.2)  1 (0.2) |
| Renal and urinary disorders  proteinuria | 2 (40) | 2 (0.4) |
| Respiratory disorders  dyspnea  nasal congestion | 1 (20)  1 (20) | 2 (0.4)  1 (0.2) |
| Skin disorders  rash  pruritis  oral ulcers | 2 (40)  1 (20)  1 (20) | 2 (0.4)  2 (0.2)  1 (0.2) |
